# Low-Field and Portable MRI for Acute Ischemic Stroke: A Systematic Review

**DOI:** 10.3390/brainsci16080788

**Published:** 2026-07-27

**Authors:** Rachana R. Borkar, Sai Dhanush Reddy Jeggari, Kamal Kandel, Kaviya Partheepan, Nishant Sharma, Sandeep Samethadka Nayak

**Affiliations:** 1 Department of Radiology and Biomedical Imaging, Yale University School of Medicine, New Haven, CT 06510, USA; rachana.borkar@yale.edu; 2 Department of Diagnostic Radiology, Yale New Haven Health Bridgeport Hospital, Bridgeport, CT 06610, USA; dreddy24797@gmail.com (S.D.R.J.); kamalkandel010@gmail.com (K.K.); 3 Trumbull High School, Trumbull, CT 06611, USA; pkav824@gmail.com; 4 Department of Internal Medicine, Yale New Haven Health Bridgeport Hospital, Bridgeport, CT 06610, USA; nishant23.med@gmail.com

**Keywords:** acute ischemic stroke, transient ischemic attack, portable MRI, low-field MRI, point-of-care imaging, neuroimaging, diagnostic accuracy

## Abstract

**Highlights:**

**What are the main findings?**

- Portable MRI enabled safe bedside imaging in emergency and intensive care settings, although detection of very small (<5–6 mm) ischemic lesions remained less reliable than conventional high-field MRI.
- Diagnostic performance was influenced by lesion size and field strength, with low-field MRI reliably detecting most clinically relevant infarcts.

**What are the implications of the main findings?**

- Low-field and portable MRI can expand timely access to stroke imaging where conventional MRI is unavailable, delayed, or unsafe, particularly in emergency departments, ICUs, and resource-limited settings.
- Continued improvements in hardware, imaging sequences, and multicenter validation studies are needed before low-field MRI can be adopted as a routine alternative to conventional high-field MRI for acute stroke evaluation.

**Abstract:**

Background: Magnetic resonance imaging (MRI) has a central role in acute ischemic stroke (AIS) and transient ischemic attack (TIA) diagnosis; however, conventional high-field MRI remains limited by infrastructure requirements, patient transport, and restricted accessibility. Low-field and portable MRI systems have emerged as potential solutions for point-of-care neuroimaging in emergency, intensive care, and resource-limited settings. Methods: A systematic review was conducted according to PRISMA 2020 guidelines. PubMed, Scopus, Web of Science, and Cochrane Library databases were searched from inception through May 2026. Studies evaluating low-field or portable MRI systems (≤0.55 T) in adults with AIS, TIA, sub-acute ischemic stroke, or suspected stroke were included. Diagnostic accuracy, feasibility, safety, workflow, and clinical utility outcomes were extracted. Risk of bias was assessed using QUADAS-2. Results: Eleven studies encompassing portable and low-field MRI platforms ranging from 0.064 T to 0.55 T were included. Portable MRI demonstrated feasibility in bedside ICU and emergency department settings without major device-related adverse events. Diagnostic performance varied by field strength, lesion size, and imaging protocol. Conclusions: Low-field and portable MRI show promising diagnostic potential for AIS and TIA, particularly when conventional MRI is unavailable, delayed, or impractical. However, current evidence is limited by small, predominantly single-center studies with substantial risk of bias, and further prospective multicenter validation is required before these technologies can be incorporated into routine clinical decision-making.

## 1. Introduction

Time is a significant factor in stroke care; however, it is not sufficient alone, as therapeutic decisions are dependent on imaging ability to detect hemorrhage, confirm ischemic injury, differentiate mimics, and estimate lesion age before the treatment window closes [1]. The Global Burden of Disease 2021 study reports more than 90 million prevalent strokes and 11.9 million incident strokes, with 7.3 million deaths in 2021. Ischemic stroke accounts for most incident events [2]. In this setting, imaging is not a passive confirmatory test. It is the step that determines the treatment pathway, whether a patient enters a thrombolysis pathway, a thrombectomy pathway, secondary prevention, observation, or an alternative diagnostic workup.

Radiological findings in the process of making decisions are crucial, using MRI to help distinguish between patients with intracerebral hemorrhage (ICH), acute ischemic stroke (AIS), transient ischemic attack (TIA), and stroke-like symptoms, especially in patients with minor deficits, posterior circulation symptoms, or nondiagnostic computed tomography (CT). Also, it helps in detecting restricted water motion early after vessel occlusion with diffusion-weighted imaging (DWI) and is more sensitive than non-contrast CT for small or hyperacute infarction. Fluid-attenuated inversion recovery (FLAIR) offers further insights; if DWI is positive and FLAIR remains negative, the lesion is more likely to be recent; this principle is used in MRI-guided thrombolysis for wake-up or unknown-onset stroke [3].

The problem is access. Conventional 1.5 Tesla (T) and 3 T MRI systems require shielded rooms and controlled safety zones, dedicated technologists, and transport to an imaging suite. These requirements are feasible for stable patients in well-resourced hospitals but are much less compatible with emergency departments (EDs), intensive care units (ICUs), mobile stroke environments, and low-resource settings. Patient transport can interrupt monitoring, ventilation, infusions, dialysis, and nursing care. CT remains indispensable because it is fast and widely available, but CT is comparatively insensitive for early ischemic change and small infarcts, a limitation that was evident in the large 0.23 T ED cohort by Suo et al., where CT detected only 122 of 304 low-field DWI-positive lesions imaged within 24 h [4].

Low-field and portable MRI systems seek to change where MRI can occur. Permanent magnets, lower fringe fields, open geometries, electromagnetic interference mitigation, and standard electrical requirements allow scanners to move closer to the patient rather than moving the patient to the scanner [5,6]. This systematic review evaluates the diagnostic performance, feasibility, safety, technical evolution, and clinical utility of portable or low-field MRI at ≤0.55 T for adults with acute stroke and TIA.

## 2. Methods

### 2.1. Protocol and Reporting Standards

We conducted this systematic review and meta-analysis in accordance with the Preferred Reporting Items for Systematic Reviews and Meta-Analyses (PRISMA) 2020 statement and the Cochrane Handbook for Systematic Reviews [7]. The review protocol was prospectively registered with the International Prospective Register of Systematic Reviews (PROSPERO) (Registration No. CRD420261433717) [8,9]. As this research was a secondary analysis of published studies, ethical approval and patient informed consent were not required.

### 2.2. Search Strategy

A comprehensive search across PubMed, Web of Science, Scopus, and the Cochrane Library from inception to May 2026 was performed. Search terms combined concepts for stroke, TIA, low-field MRI, portable MRI, point-of-care MRI, diffusion-weighted imaging, and FLAIR. Reference lists, trial registries, and recent reviews of portable low-field MRI were also screened. Full search strategies are provided in Supplementary Table S1.

### 2.3. Eligibility Criteria

Eligibility was framed by population, index test, reference standard, and outcomes. Individual adults aged 18 years or older, with suspected or confirmed AIS, TIA, minor ischemic stroke, stroke mimic, or mixed acute and subacute stroke presentation were included, with emphasis on patients imaged within 72 h of symptom onset or last known well. ED, ICU, inpatient stroke unit, mobile imaging, and resource-limited settings were eligible.

The index test was low-field portable MRI with a field strength of ≤0.55 T, regardless of magnet geometry, manufacturer, sequence package, or software version. Eligible systems included 0.064 T portable MRI, 0.23 T mobile MRI, 0.35 T open MRI, 0.55 T MRI, and field-cycling platforms operating across ultra-low and low magnetic fields. Reference standards were conventional high-field MRI at ≥1.5 T or CT scans. Outcomes included sensitivity, specificity, accuracy, positive predictive value (PPV), negative predictive value (NPV), lesion-size detection thresholds, DWI-FLAIR mismatch, AIS-ICH differentiation, lesion volume agreement, interrater reliability, scan duration, technical failure, safety, patient tolerance, operator requirements, and clinical decision impact. We excluded case reports and case series with fewer than 10 participants, animal-only studies, technical papers without diagnostic or clinically relevant imaging outcomes, non-stroke populations, narrative reviews, editorials, commentaries, and non-English studies.

### 2.4. Study Selection and Data Extraction

Two reviewers independently screened titles, abstracts, and full texts. Disagreements were resolved by consensus or by a third reviewer. Data extraction used a predefined form. Extracted variables included study identification, country, setting, design, enrollment period, funding, conflicts of interest, registration or ethics approval, sample size, target condition, age, sex, National Institutes of Health Stroke Scale score, symptom-to-scan timing, inclusion and exclusion criteria, field strength, scanner model, hardware and software version, sequences, DWI parameters, acquisition time, post-processing, operator training, contraindications, reference standard, reader blinding, index-reference interval, diagnostic outcomes, feasibility outcomes, adverse events, and clinical decision effects.

### 2.5. Risk of Bias

Risk of bias and applicability were assessed with the modified Quality Assessment of Diagnostic Accuracy Studies (QUADAS-2) tool across patient selection, index test, reference standard, and flow/timing domains [10].

## 3. Results

### 3.1. Study Selection

**Figure 1:**
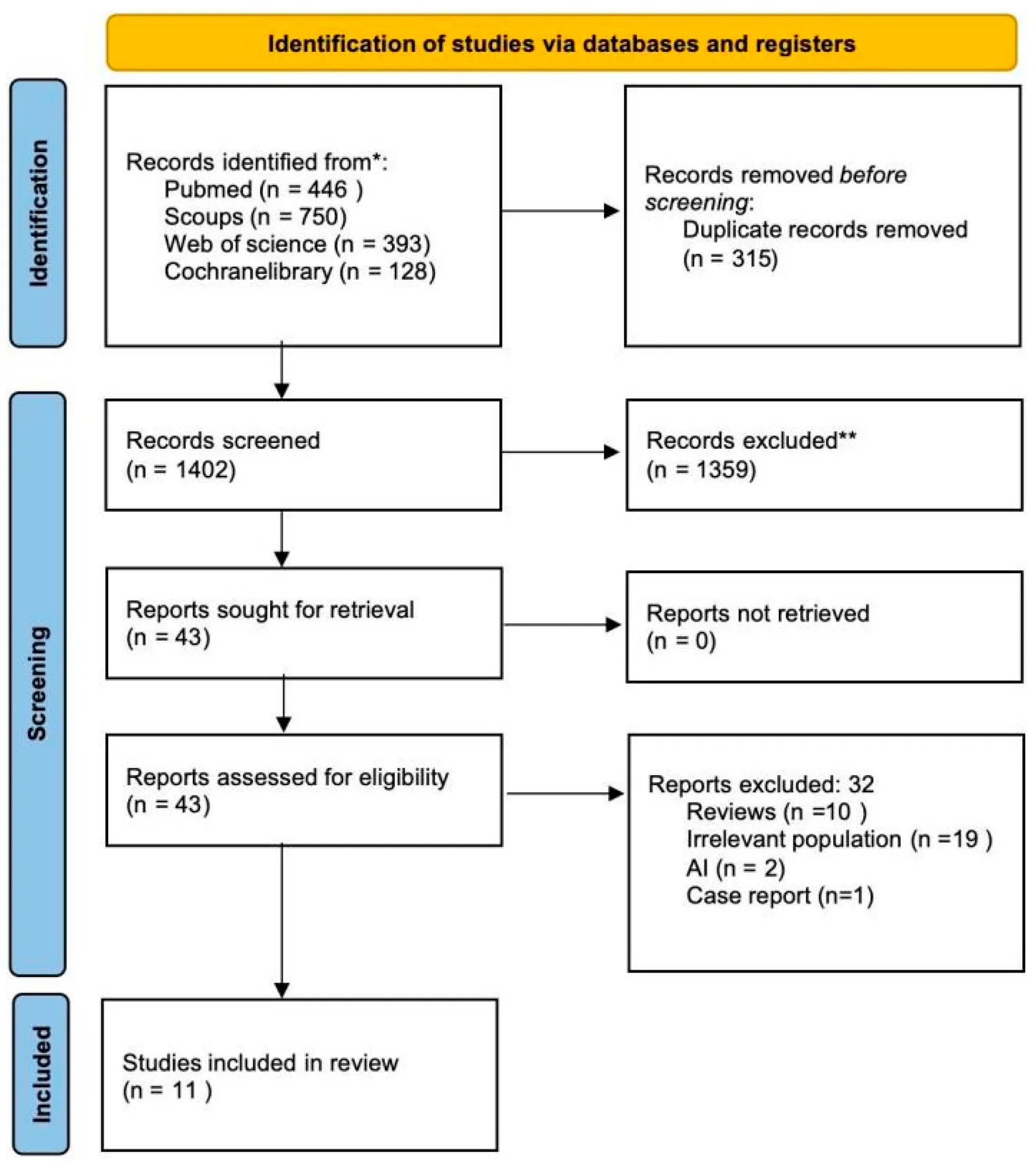
PRISMA flow diagram [11].

The search yielded 1717 records. After removing 315 duplicates, 1402 records proceeded to title and abstract screening. Of these, 1359 were excluded. The extracted full-text included 11 eligible studies published between 2005 and 2026. They describe the evolution of low-field stroke MRI from early open low-field imaging and field-cycling proof-of-concept work to bedside portable MRI and mobile ED systems. The final PRISMA diagram is shown in Figure 1.

### 3.2. Study Characteristics

The studies covered five technology groups. Five studies evaluated 0.064 T Hyperfine portable MRI in the ICU, ED, acute stroke, DWI-FLAIR mismatch, and optimized DWI settings (Sheth 2021; Yuen 2022; Sorby-Adams 2024; von Danwitz 2025; Sorby-Adams 2026) [12,13,14,15,16]. Three studies evaluated 0.23 T ACUTA Elfin/Ray Plus mobile MRI for minor stroke/TIA diagnosis or AIS-ICH differentiation (Suo 2024; Suo 2026; Xie 2024) [4,17,18]. One study evaluated a 0.35 T open MRI within 3 h of symptom onset (Wohlgemuth 2005) [19]. One prospective comparison studied 0.55 T MRI against 1.5 T MRI (Rusche 2022) [9]. One field-cycling study examined endogenous T1 dispersion contrast in subacute ischemic stroke (Mallikourti 2024) [20]. Clinical settings varied substantially. Sheth et al. and Yuen et al. focused on bedside imaging in critically ill or hospitalized patients, whereas Suo et al. studied mobile 0.23 T MRI in ED minor stroke/TIA pathways. Reference standards ranged from 1.5 T or 3 T MRI to CT and the final clinical radiological diagnosis. Sample sizes ranged from small pilot cohorts to the 974-patient ED study by Suo et al., as shown in Table 1.

### 3.3. Risk of Bias and Applicability

The overall quality of the included studies exhibited a high risk of bias. High risk of bias was most frequently observed in the patient selection domain and overall risk of bias assessment (81.8%), primarily due to non-consecutive enrollment and selective inclusion criteria. In contrast, the index test domain demonstrated predominantly low risk of bias, while applicability concerns were high across patient selection and overall applicability domains, as shown in Supplementary Table S2 and Figure 1.

### 3.4. Technology and Field Strength

At 0.064 T, portable MRI consistently showed that bedside brain imaging is feasible, but diagnostic performance depended on lesion size, sequence design, and hardware generation. In the 50-patient ischemic stroke cohort by Yuen et al., portable MRI detected infarcts in 45 patients; sequence-level sensitivity was 98% for T2-weighted imaging, 100% for FLAIR, and 86% for DWI, and missed lesions were 4–10 mm [13]. The European acute stroke pilot by von Danwitz et al. found a sensitivity of 67% and a specificity of 100% for ischemic lesion detection compared with high-field MRI; all missed lesions were smaller than 6 mm, while lesions ≥6 mm were detected [15]. DWI-FLAIR mismatch was also measurable at low field. In Sorby-Adams et al., visual mismatch identified patients imaged within 4.5 h with 60% sensitivity and 82% specificity; a quantitative FLAIR signal intensity ratio <1.15 improved these estimates to 70% and 85.2%, respectively [16].

The latter optimized DWI study suggests that technical refinement matters as much as field strength. In patients with AIS and mimics, single-direction 0.064 T DWI yielded a sensitivity of 78.2% and a specificity of 96.8%; multidirectional DWI improved sensitivity to 95% and specificity to 100%. Next-generation hardware shortened acquisition by approximately 30% and detected lesions as small as 0.15 mL [14]. These findings indicate a moving target: pooled results from early hardware probably underestimate current performance.

At 0.23 T, diagnostic performance was strongest in minor stroke/TIA and hemorrhage differentiation. In a 102-patient paired comparison with 3 T MRI, mobile 0.23 T MRI achieved 97.9% sensitivity and 96.1% accuracy; missed lesions were subcortical or brainstem infarcts smaller than 5 mm [17]. In the 974-patient ED cohort, low-field DWI detected acute infarction in 338 patients with minor stroke/TIA, while CT detected only 122 of 304 low-field-positive lesions among those who underwent CT within 24 h [4]. Xie et al. tested a hemorrhage-emphasized inversion recovery sequence and found complete differentiation of AIS from ICH in 60 patients within 24 h, with 100% sensitivity, 100% specificity, and complete interreader agreement [18].

Older low-field studies add context. The 0.35 T open MRI study detected early infarction with 94% DWI sensitivity within 3 h, compared with 73% for CT, although motion artifacts required repeated DWI in 7 of 18 patients and sedation in four [18]. At 0.55 T, Rusche et al. reported a sensitivity of 92.9% and a specificity of 100% for the most experienced reader compared with 1.5 T MRI [9]. Field-cycling MRI did not provide standard accuracy estimates, but it showed substantial reader agreement and stronger infarct-to-contralateral contrast at lower fields [20].

### 3.5. Feasibility, Safety, and Workflow

Feasibility findings were clinically meaningful. A bedside portable MRI was performed in ICU environments without removing major supportive equipment, and no major device-related adverse events were reported [12,13]. The 0.23 T ED workflow was comparatively brief: Suo et al. reported a 10-min 28-s protocol, and Xie et al. reported combined DWI and HEIR acquisition in 5 min 51 s [4,18]. The main trade-off was image quality: longer protocols and motion susceptibility improved yield in some cases but reduced workflow simplicity.

## 4. Discussion

This review suggests that low-field MRI has demonstrated encouraging technical feasibility and diagnostic potential for selected stroke applications. However, the available evidence remains limited by small sample sizes, single-center study designs, and methodological heterogeneity. Through included studies, low-field MRI detects significantly clinically relevant ischemic lesions, differentiates AIS from ICH in early 0.23 T data, sustains DWI-FLAIR mismatch assessment, and permits bedside imaging in patients for whom transport is undesirable or unsafe.

The most reproducible limitation is lesion size. Suo et al. missed subcortical or brainstem lesions smaller than 5 mm at 0.23 T [17]. In addition, Von Danwitz et al. missed four high-field MRI-positive infarcts at 0.064 T, any lesion smaller than 6 mm [15]. Also, a study by Yuen et al. reported undetected lesions in the 4–10 mm range [12]. Hence, low-field MRI should be interpreted as a rule-in tool in many settings rather than a definitive rule-out test, especially for brainstem, cerebellar, thalamic, or deep white matter symptoms. Previous reviews demonstrated that reduced infrastructure, lower power requirements, and bedside compatibility can extend access beyond the conventional MRI suite [5]. The stroke literature emphasizes that low-field MRI can identify DWI-positive infarction, show FLAIR evolution, quantify lesion volume, and differentiate hemorrhage in selected protocols. Compared with CT, it offers tissue-based early ischemia detection; compared with high-field MRI, it trades away spatial resolution, signal-to-noise ratio, and vascular/perfusion capability.

The DWI-FLAIR mismatch data are particularly relevant. High-field MRI-guided thrombolysis in unknown-onset stroke depends on the biological relationship between DWI positivity and delayed FLAIR hyperintensity [21]. Sorby-Adams et al. show that this relationship is visible at 0.064 T, but the performance is not yet strong enough to treat low-field mismatch as interchangeable with high-field mismatch [16]. The better role at present may be triage: low-field MRI can identify patients who may benefit from urgent high-field MRI, transfer, or expert review when conventional imaging is delayed.

The ED use case is clearest for minor stroke and TIA. These patients often have nondiagnostic CTs and management decisions that depend on whether symptoms represent tissue injury. In the large Suo et al. cohort, 0.23 T DWI found acute infarction in a substantial proportion of minor stroke/TIA presentations, while CT missed most low-field-positive lesions among those scanned with CT [4]. These findings suggest that mobile low-field MRI may complement CT in selected patients with minor stroke or TIA, although prospective studies evaluating workflow integration and patient outcomes are still needed. For ICU patients, the value is different. Bedside MRI may reduce transport hazards, particularly in ventilated or hemodynamically unstable patients. Sheth et al. demonstrated feasibility in critical illness, and Yuen et al. showed that portable MRI could detect ischemic lesions and follow lesion evolution [12,13]. In many ICU scenarios, the key question is whether a new territorial infarct, hemorrhage, edema, or evolution changes management. Low-field MRI may also have a role in resource-limited and prehospital systems, but portability alone is insufficient. A useful service still requires trained operators, maintenance, image transfer, timely interpretation, clinical protocols, and access to vascular imaging when large vessel occlusion is suspected.

Field strength influences image quality, but the included studies show that performance is not determined by field strength alone. Sequence design, DWI directionality, gradient performance, reconstruction, denoising, motion correction, and hardware generation all matter. The optimized DWI study by Sorby-Adams et al. is the clearest example: multi-direction DWI and newer hardware improved sensitivity and reduced acquisition time on the same 0.064 T platform [14]. The 0.23 T HEIR sequence similarly shows how sequence design can target a specific clinical need, in this case, AIS-ICH differentiation [18].

Artificial intelligence-based reconstruction may further improve low-field MRI, particularly where signal-to-noise ratio and lesion conspicuity are limiting factors. Stroke-Aware CycleGAN reportedly improved lesion-volume correlation with high-field DWI from R = 0.462 to R = 0.852 in paired scans [22]. This direction is promising, but it demands prospective validation. Enhancement methods must boost diagnosis without increasing the rate of false lesions, suppressing subtle infarcts, or shifting treatment decisions without evidence.

This review integrates diagnostic accuracy, technical evolution, and workflow evidence rather than treating low-field MRI as a single device class. It also investigates ED diagnosis, ICU feasibility, DWI-FLAIR mismatch, AIS-ICH differentiation, and lesion-size limits, which are important because these are different clinical questions.

The review also inherits limitations from the primary literature. Studies are single-center, small, or proof-of-concept. Enrollment often excludes patients who may benefit most from bedside imaging, including those with severe deficits, agitation, impaired consciousness, or device-related concerns. Reference standards vary, and long index-reference intervals may change lesion visibility. Rapid hardware and software evolution also limits temporal generalizability.

The next phase should move from feasibility to implementation-quality evidence. Multicenter diagnostic studies should use standardized reference MRI, prespecified lesion-size strata, and blinded reading. Future trials should include posterior circulation symptoms, aphasia, agitation, cardiac implantable electronic devices when permitted, and ICU-level illness. Also, studies should report who performs the scan, who interprets it, how fast results reach the team, and what decision changes afterward. Cost-effectiveness studies should measure scanner cost, staffing, room requirements, maintenance, transfer decisions, avoided transport, treatment changes, and equity effects. Technical work should continue on low-field angiography, perfusion, motion correction, multi-direction DWI, and validated enhancement pipelines.

## 5. Conclusions

Low-field and portable MRI show clinically meaningful promise for acute stroke and TIA care, particularly when conventional MRI is delayed, unavailable, or unsafe. Current evidence suggests potential applications for ischemic lesion detection, bedside ICU imaging, and selected imaging tasks such as AIS–ICH differentiation and DWI–FLAIR assessment. However, these findings should be considered preliminary because they are derived predominantly from small, single-center studies with an overall high risk of bias. Larger prospective multicenter studies are required before routine clinical implementation can be recommended.

## Figures and Tables

**Table 1:** Summary of the included studies. Abbreviations: AIS = Acute ischemic stroke, AMS = Altered mental status, CTA = Computed tomography angiography, CTP = Computed tomography perfusion, CTV = Computed tomography venography, DWI = Diffusion-weighted imaging, FLAIR = Fluid-attenuated inversion recovery, GRE = Gradient recalled echo, ICH = Intracerebral hemorrhage, MIS = Minor ischemic stroke, MRI = Magnetic resonance imaging, NIHSS = National Institutes of Health Stroke Scale, SAH = Subarachnoid hemorrhage, SWI = Susceptibility-weighted imaging, TIA = Transient ischemic attack.

Study (Author, Year)	Total Sample (N)	Target Condition	Age (Years)	Female (%)	Inclusion Criteria	Exclusion Criteria	Stroke Subtype Distribution	Field Strength	Reference Modality	MRI/Imaging Sequences
Suo et al., 2024 [15]	102	MIS or TIA with acute/early subacute ischemic lesions	Median: 60	15.7	Consecutive patients undergoing both mobile and 3 T fixed MRI within 14 days	-	MIS/TIA cohort; 4 missed infarcts <5 mm	0.23 T	3 T fixed MRI	T1-FLAIR, T2-FLAIR, T2 fast spin-echo, DWI with ADC
Suo et al., 2026 [4]	974	MIS (NIHSS ≤ 5) or TIA within 72 h	Median 64 (DWI+)	30.9	ED patients with MIS/TIA and focal deficits	>72 h onset, hemorrhage, NIHSS > 5	Acute infarction 338/974	0.23 T	NCCT ± multimodal CT	NCCT, CTA, CTV, CTP
von Danwitz et al., 2025 [15]	17	Suspected ischemic stroke within 72 h	Median: 76	41.2	Adults > 18 years within 72 h	Consent inability, claustrophobia, device contraindications	12 ischemic lesions; 3 mimics	0.064 T	1.5 T/3 T HF-MRI; CT	DWI, FLAIR, T2 GRE
Sheth et al., 2021 [12]	50	Stroke	Mean: 59	23	Neurological injury without MRI contraindications	Large body habitus	Stroke, SAH, TBI, tumors, COVID AMS	0.064 T	Conventional CT/MRI	-
Rusche et al., 2022 [9]	27	Suspected stroke/TIA	Mean: 71	44	Patients undergoing 1.5 T MRI followed by 0.55 T MRI	Poor-quality datasets, incompatible implants	17 stroke; 10 controls	0.55 T	1.5 T MRI	DWI/ADC, FLAIR, SWI
Mallikourti et al., 2024 [20]	14	Ischemic stroke	Mean: 62	0	Documented ischemic stroke within 7 days	Prior stroke, BMI > 28	Subacute ischemic stroke only	0.2 mT–0.2 T	CT and/or 3 T MRI	T1, T2, FLAIR, DWI, T2 GRE
Sorby-Adams et al., 2024 [16]	71	Acute ischemic stroke within 24 h	Mean: 71	49	Adults with AIS confirmed on MRI	Pregnancy, implants, hemorrhagic transformation	AIS only	0.064 T	1.5 T–3 T MRI	HF-DWI with ADC; HF-FLAIR
Yuen et al., 2022 [13]	50	Confirmed ischemic stroke	Median: 61	46	NICU/ED/COVID ICU patients	MRI contraindications	MCA territory most common	0.064 T	SOC NCCT or MRI	SOC MRI DWI and CT
Sorby-Adams et al., 2026 [14]	95	Suspected AIS	AIS Mean: 67	36	Adults with suspected AIS	ICH on CT, implants, motion artifact	62 AIS; 33 mimics	0.064 T	1.5 T/3 T MRI	Conventional DWI
Xie et al., 2024 [18]	60	AIS vs. ICH differentiation	Median: 60	23.3	Confirmed AIS or ICH within 24 h	Not fully reported	30 AIS; 30 ICH	0.23 T	CT for ICH; 3 T MRI for AIS	CT and diagnostic MRI
Wohlgemuth et al., 2005 [19]	18	Acute focal cerebral ischemia within 3 h	Mean: 58.5	31.6	Candidates for thrombolysis within 3 h	Age > 75, MRI contraindications	15 infarction; 3 ICH	0.35 T	CT plus follow-up MRI	T2, DWI

## Data Availability

No new data were created or analyzed in this study.

## Supplementary Materials

The following supporting information can be downloaded at: https://www.mdpi.com/article/10.3390/brainsci16080788/s1, Figure S1: Detailed search strategy; Table S1: Detailed search strategy; Table S2: Quality Assessment of Diagnostic Accuracy Studies-2 assessment result.
