# Supplementary material for: Low-Field and Portable MRI for Acute Ischemic Stroke: A Systematic Review
Source: Brain Sci. 2026 Jul 27;16(8):788. doi: 10.3390/brainsci16080788 (PMC13511266; doi:10.3390/brainsci16080788)

Low-Field and Portable MRI for Acute Ischemic Stroke

Authors:Rachana R. Borkar, M.D.[1]; Sai Dhanush Reddy Jeggari, MBBS.[2]; Kamal Kandel, M.D.[2]; Kaviya Partheepan [3]; Nishant Sharma, M.D.[4]; Sandeep Samethadka Nayak, M.D.[5]

Supplementary Tables:

Supplementary Table S1: Detailed search strategy

Supplementary Table S2. QUADAS assessment for each study

Supplementary Figures:

Supplementary Figure S1. Quality Assessment of Diagnostic Accuracy Studies-2 assessment result

Supplementary Table S1: Detailed search strategy

Database	Search	strategy
Pubmed	("Stroke" OR "Ischemic Stroke" OR "acute stroke" OR "brain infarction" OR "cerebral infarction" OR "cerebral ischemia" OR "brain ischemia" OR "acute ischemic stroke" OR AIS) AND ("Magnetic Resonance Imaging" OR MRI) AND ("low field" OR "ultra-low field" OR "portable" OR "bedside" OR "point of care" OR "mobile MRI" OR "<0.5T" OR "low magnetic field" OR Hyperfine OR Swoop OR "low field strength" OR "handheld" OR "compact" OR	446
Scopus		750
WOS		393
Cochrane		128

	"transportable" OR "deployable")	

Supplementary Table S2. QUADAS assessment for each study

Study ID	Risk of Bias					Applicability Concern			
	Patient Selection	Index Test	Reference Standard	Flow and Timing	Overall ROB	Patient Selection	Index Test	Reference Standard	Overall applicability
Suo et al. (2024)	Unclear	Low	Low	Low	Unclear	Low	Low	Low	Low
Suo et al. (2026)	Low	Low	High	High	High	Low	Low	High	High
von Danwitz et al. (2025)	High	Low	Low	Unclear	High	High	Low	Low	High
Sheth et al. (2021)	High	Unclear	High	High	High	High	Low	High	High
Rusche et al. (2022)	High	Low	Low	Low	High	High	Low	Low	High
Mallikourti et al. (2024)	High	Unclear	Unclear	High	High	High	Low	High	High
Sorby-Adams et al. (2024)	High	Low	Low	Unclear	High	High	Low	Low	High
Yuen et al. (2022)	High	Unclear	High	High	High	High	Low	High	High
Sorby-Adams et al. (2026)	Unclear	Low	Low	Unclear	Unclear	Low	Low	Low	High
Xie et al. (2024)	High	Low	Low	Low	High	High	Low	Low	High

Wohlgemuth et al. (2005)	Unclear	Unclear	High	Unclear	High	Low	Low	High	High

Supplementary Figure S1. Quality Assessment of Diagnostic Accuracy Studies-2 assessment result

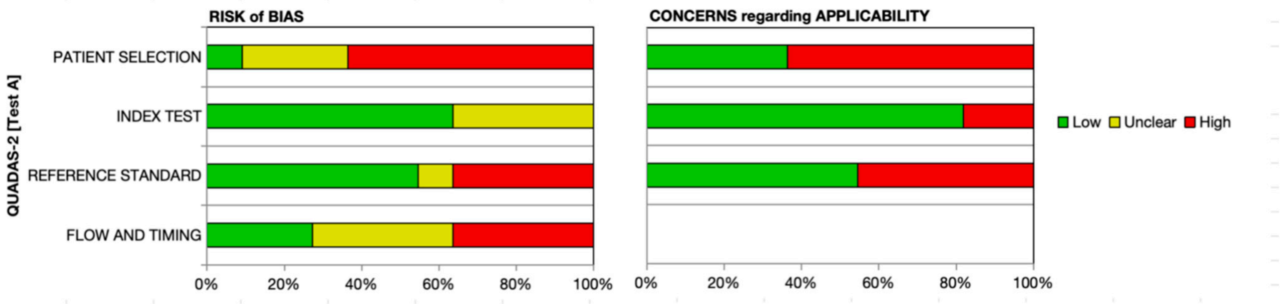

Supplement: Supplementary file 1 [file brainsci-16-00788-s001.zip › Supplementary materials.pdf]
